# Nanoparticles as Potential Novel Therapies for Urinary Tract Infections

**DOI:** 10.3389/fcimb.2021.656496

**Published:** 2021-04-19

**Authors:** Sofía V. Sánchez, Nicolás Navarro, Johanna Catalán-Figueroa, Javier O. Morales

**Affiliations:** ^1^ Department of Pharmaceutical Science and Technology, School of Chemical and Pharmaceutical Sciences, University of Chile, Santiago, Chile; ^2^ Center of New Drugs for Hypertension (CENDHY), Santiago, Chile; ^3^ Advanced Center for Chronic Diseases (ACCDiS), Santiago, Chile; ^4^ Departamento Farmacología, Facultad de Ciencias Químicas, Instituto de Farmacología experimental de Córdoba (IFEC-CONICET), Universidad Nacional de Córdoba, Córdoba, Argentina; ^5^ Facultad de Medicina, Escuela de Química y Farmacia, Universidad Católica del Maule, Talca, Chile

**Keywords:** urinary tract infection therapies, composite materials, antibacterial, antibiofilm, organic nanoparticles, inorganic nanoparticles

## Abstract

Urinary tract infection (UTI) is one of the most common reasons for antibiotic treatment. Nevertheless, uropathogens are steadily becoming resistant to currently available therapies. In this context, nanotechnology emerges as an innovative and promising approach among diverse strategies currently under development. In this review we deeply discuss different nanoparticles (NPs) used in UTI treatment, including organic NPs, nanodiamonds, chemical and green synthesized inorganic NPs, and NPs made of composite materials. In addition, we compare the effects of different NPs against uropathogens *in vivo* and *in vitro* and discuss their potential impact the in the near future.

## Introduction

Urinary tract infection (UTI) is the second most common infection in humans (Foxman, 2014; Jarzembowski et al., 2018), affecting around 150 million people each year around the world. It possesses a public health cost estimated in around six billion dollars (treatment and healthcare), representing a significant economic and public health burden (Stamm and Norrby, 2001).

UTI can be acquired in the community and hospitals (Flores-Mireles et al., 2015; Rawal et al., 2019), and is defined as an inflammatory response of the urothelial cells, which is induced by pathogenic microorganisms (Gram-negative and Gram-positive bacteria, and fungi) that are capable of colonizing any urinary tract tissue (urethra, bladder, ureters and kidneys) (Foxman, 2014; Jarzembowski et al., 2018), being uropathogenic *Escherichia coli* (UPEC) the most frequent agent of the infection. Additionally, other important pathogens are *Klebsiella pneumoniae, Staphylococcus saprophyticus, Enterococcus faecalis, group B Streptococcus, Proteus mirabilis, Pseudomonas aeruginosa, Staphylococcus aureus and Candida sp* (Flores-Mireles et al., 2015). Particularly, in hospitals, catheter-associated UTI (CAUTI) is one of the most common nosocomial infections, and it can lead to increased morbidity and mortality due to secondary bloodstream infections (Flores-Mireles et al., 2015).

Historically, UTI treatment begin with empiric antibiotics, such as nitrofurantoin, fosfomycin, ciprofloxacin, or levofloxacin. Nevertheless, with the constant emergence of multi-drug-resistant (MDR) pathogens, the treatment now begins with β-lactams, fluoroquinolones, trimethoprim-sulfamethoxazole, amoxicillin-clavulanic acid or a third-generation cephalosporin, until the acquisition of culture sensitivity results (Flores-Mireles et al., 2015; Klein and Hultgren, 2020). In addition, these bacteria can also form biofilms, which usually result in difficult-to-treat infections, causing long-term hospitalizations and high health care costs (Flores-Mireles et al., 2015; Jamal et al., 2018; Vestby et al., 2020). Biofilms are clusters of microorganisms embedded in an extracellular matrix produced by themselves (Jamal et al., 2018; Vestby et al., 2020); they are formed when the microorganisms adhere in clusters to each other or/and to a living or non-living surface, developing well-structured micro-colonies, that after maturation, end up with detachment (Flores-Mireles et al., 2015; Jamal et al., 2018; Vestby et al., 2020). Moreover, the biofilm also functions as a host-defense mechanism for the bacteria, and it enhances antibiotic tolerance.

Thus, the constant emergence of antibiotic-resistant uropathogens, along with their ability to develop biofilms, hinders infection treatment. For this reason, the development of new effective treatment strategies to prevent and combat both, UTI and CAUTI efficiently, is a challenge for science (Flores-Mireles et al., 2015; Rawal et al., 2019). In recent years, nanotechnology has had an important impact on drug discovery and development of more effective and potent medicine. Nanoparticles (NPs) are complex three-dimensional structures, with a diameter up to 1000 nm, which are designed to elicit multiple effects. The properties of NPs are associated with their reduced size and the interplay between their components and the environment (Rawal et al., 2019). In nanomedicine, NPs components have diverse functions, since not only the active ingredient elicit therapeutic effects, but also excipient may exhibit diverse advantageous properties. For example, they can highly improve drug delivery into specific sites (i.e.: tissues or target cells) by: enhancing drug solubility; improving drugs penetration across biological barriers; and controlling drug release, thus enhancing bioavailability (Slevin, 2012; Rawal et al., 2019). Therefore, based on the advantages NPs exhibit, they can be used to design and develop different drug delivery systems. Examples of these systems for UTI management include organic and inorganic nanoparticles (and their combinations); the design of these NPs combine a matrix with antimicrobial characteristics and the addition of active molecules (such as antibiotics or proteins), to improve the antibacterial effect of the new systems (Mittal et al., 2018). Thus, the goal of this review is to introduce and discuss the recent nanotechnological advances to improve UTI treatment. We focus on different systems and materials used for NPs design and synthesis, and on the results that these NPs have shown against the uropathogenic microorganisms.

## Organic Nanoparticles

Organic NPs are stable particles consisting of organic compounds like polymers or lipids, that have found impressive applications in drug delivery (Mitragotri and Stayton, 2014). Therefore, due to their composition they are more suited for biological applications and have gained more importance every day in nanomedicine and pharmaceutical industry (Mitragotri and Stayton, 2014). In the search for UTIs new treatment strategies, they are considered primarily as nanocarriers (Mitragotri and Stayton, 2014; Mittal et al., 2018) (**Table 1**). These nanocarriers maintain special relevance given their inhibitory activity over pathogens’ biofilm formation. In this context, Macedo et al., observed that the functionalization of standard urinary catheters material (i.e.: polydimethylsiloxane) with antibacterial aminocellulose nanospheres (ACNSs), provided a growth inhibitory activity of 80% against *E. coli-*associated biofilm formation, compared to the control silicone surface (Macedo et al., 2017). In addition, Franceso et al., evaluated the effect of urinary catheters functionalized with ACNSs and hyaluronic acid (HA) polyanion to develop a layer-by-layer construct on silicone surfaces against *P. aeruginosa* biofilm. The results determined that a 5-layer construct reduced the number of bacteria clusters. Moreover, the 10-layer construct exhibited antibiofilm activity, without signs of bacteria colonization (Francesko et al., 2016).

**Table 1:** Physicochemical characteristics of organic nanoparticles.

Organic NPs	REF	Functional group		Diameter (nm)	ζ - potential (mV)	Tested uropathogens	Activity

Aminocellulose nanospheres	(Macedo et al., 2017)	Primary amine		268 ± 7	103 ± 2	*E. coli*	Antibiofilm
Aminocellulose nanospheres/hyaluronic acid construct	(Francesko et al., 2016)	Hyaluronic acid		268 ± 7	103 ± 2	*P. aeruginosa*	Antibiofilm
Glycerol monolaurate nanocapsules	(Lopes et al., 2019)	–		190.7 ± 2	-23.3 ± 3	*P. aeruginosa*	Antibiofilm
Chlorhexidine-loaded poly(ε-caprolactone) nanospheres	(Phuengkham and Nasongkla, 2015)	poly(ε-caprolactone)		198.8 ± 6.9	–	*S. aureus, S. epidermidis* and *E. coli*	Antibacterial
Chlorhexidine-loaded poly(ε-caprolactone) nanospheres	(Srisang and Nasongkla, 2019)	poly(ε-caprolactone)		152 ± 37	40.21 ± 1.08	*E. coli, S. aureus*, and *C. albicans*	Antibacterial
Polyurethane chlorhexidine nanocomposites	(Francesko et al., 2016)	–		–	–	*S. epidermidis*	Antibacterial
Kanamycin-chitosan nanoparticles	(Venkat Kumar et al., 2016)	NA		225	36	*E. coli* and *P. mirabilis*	Antibacterial
							Antibiofilm
Chlorin e6 encapsulated charge-conversion polymeric nanoparticles	(Lui et al., 2015)	poly (β-amino ester)s		33.5 ± 4.5	-6	*S. aureus* and *E. coli*	Antibacterial
chitosan-coated poly(lactide-co-glycolide) nanoparticles loaded with amphotericin B	(Ludwig et al., 2018)	poly(lactide-co-glycolide)		459 ± 22	18 ± 3.5	*Candida* sp.	Antifungal
Nano catechin	(Yoon et al., 2011)	–		100 – 200	–	*E. coli*	Antibacterial
Polyphenol 60 and curcumin nanoemulsion based gel (intravaginal)	(Kaur et al., 2017a)	–		211.2	-32.7	UPEC	Antibacterial
polyphenol 60 + cranberry nanoemulsion based gel (intravaginal)	(Kaur et al., 2017b)	–		58	-16	*E. coli*	Antibacterial
Polyphenol 60 and ciprofloxacin	(Atinderpal et al., 2018)	–		151.7	55.3	*E. coli, K. pneumoniae, P. mirabilis, C. amalonatus* and *C. diversus*	Antibacterial
Bilayer nanocapsules containing lutA antigen (vaccine)	(Crecente-Campo et al., 2018)	–		200 ± 2	42 ± 4	*E. coli, K. pneumoniae, P. mirabilis, C. amalonatus* and *C. diversus*	Vaccine

The antibiofilm effect of glycerol monolaurate (GML) was also evaluated against *P. aeruginosa.* GML is an emulsifier with antimicrobial properties against enveloped viruses, Gram-negative and Gram-positive bacteria (Lopes et al., 2019). In the work of Lopes et al., free GML and GML nanocapsules were evaluated; they reported that only when the biofilms were treated with GML nanocapsules, the biomass, proteins, polysaccharides, and viable *P. aeruginosa* were significantly reduced. Moreover, GML nanocapsules were able to inhibit biofilm formation by about 37% in subinhibitory concentrations (Lopes et al., 2019). These results support an antibiofilm activity of AC and GML, when loaded into NPs in comparison to free molecules, suggesting that nanotechnology can improve the antibacterial properties of some compounds. Interestingly, in the case of AC, it only exerts antibacterial effects as a nanosphere, since its antibiofilm ability depends on the high cationic charge of nanospheres surface, which favors the interaction with the negatively charged cell membrane of the bacteria (Macedo et al., 2017). In regard to GML, in its free form, its antibacterial properties cannot be fully exploited, since both, its poor solubility and low bioavailability, limit their use (Lopes et al., 2019). Therefore, these results invite to perform further investigations with these molecules, since NPs not only can be used as antibiofilm agents, but also, they could be employed for the treatment and prevention of other infections, beyond the catheter-associated nosocomial ones.

Another organic material explored for silicone surface coating is chlorhexidine (CHX). Chlorhexidine is an antiseptic agent usually used against gingivitis or to sterilize surgical instruments (Supranoto et al., 2015). Phuengkham *el al.*, observed that CHX exhibited a minimum inhibitory concentration (MIC) between 0.3 and 0.6 µg/mL against *S. aureus*, *S. epidermidis*, and *E. col*i. Next, they prepared poly(ε-caprolactone) (PCL) nanospheres loaded with CHX (CHX-NP). The aiming of PCL-based NPs was to obtained a controlled drug release and to provide uniformity and stability for the formulation (Jones, 2004). During the *in vitro* release from silicon tubes studies, they observed that CHX-NPs released 3.35 ± 0.16 µg/day of CHX for 2 weeks, whereas in the physical mixture of CHX and PCL (CHX-PCL) most of CHX was released after 4 days; both cases contained 2 mg/mL of CHX. In addition, it was shown that the use of a silicone surface coated with both, CHX-NPs and CHX-PCL, decreased the initial adherence for all tested bacteria, up to 15 and 5 days, respectively (Phuengkham and Nasongkla, 2015). Likewise, similar antimicrobial results were observed by Srisand et al., using the same CHX-NPs as a cover of foley catheters, which inhibited 100-fold higher than uncoated catheters the growth of *E. coli, S. aureus*, and *C. albicans*. It is noteworthy that these NPs can be long-term preserved by 2% (w/v) sucrose lyophilization (Srisang and Nasongkla, 2019).

Therefore, these studies suggest the multiple applications of CHX when used within nanosystems, such as enhanced antibacterial potential against UTI pathogens and the capability to achieve sustained release. All these characteristics suggest that CHX is an excellent candidate for subsequent investigations, from the prevention of CAUTI to UTI treatment.

On the other hand, in a similar surface-charge strategy, Liu et al. analyzed an antimicrobial photodynamic therapy (PDT) using Chlorin e6 (Ce6) encapsulated in charge-conversion polymeric nanoparticles (NPs). PDT is a photochemical reaction, that uses a photosensitizer (Ce6) to generate reactive oxygen species (ROS) upon laser irradiation (Patrice, 2007). PDT exhibits advantages over UTI antibiotic therapy, since the photodestruction of the photosensitizer can disrupt both, Gram-positive and negative bacteria walls at low concentrations and reduced cytotoxicity, as well as bypassing the selection of resistant strains (Liu et al., 2015). To support this concept, the authors first evaluated the antibacterial properties of NPs, free Ce6, and polymer (P1) by plate count after PDT. NPs showed a complete inhibition with a MIC of 11.93 µg/mL for *S. aureus*, and 17.91 µg/mL *for E. coli*. Ce6 exhibited higher MIC values, while no antibacterial activity was observed for P1. Additionally, in an cystitis mouse model, the authors showed that NPs administration had a significant decrease in bacteria burden in infected tissues in comparison to free Ce6 (Liu et al., 2015).

In addition, considering that fungus are also common urinary tract pathogens, Ludwig et al, evaluated how to decrease Amphotericin B (AmB) toxicity using chitosan (CS)-coated poly(lactide-co-glycolide) (PLGA) NPs (CS-NPs) (Ludwig et al., 2018). The authors evaluated the antifungal effect of AmB loaded in CS-NPs (AmB-CS-NPs) (sustained release) in 20 different strains of *Candida* sp. isolated from infected patients (Ludwig et al., 2018). This work used the European Committee on Antimicrobial Susceptibility Testing (EUCAST) (EUCAST) to determine the *in vitro* susceptibility of all of isolated from *Candida* strains. In the *in vitro* susceptibility tests, all the isolates were susceptible to both, free AmB and AmB-CS-NPs. On the other hand, in the cytotoxicity assay, AmB-CS-NPs presented negligible hemolysis, while free AmB showed 100% of hemolysis in 24h (Ludwig et al., 2018).

Natural compounds had also been widely investigated to treat UTIs using the advances in nanomedicine and nanotechnology (Talegaonkar et al., 2008). In this area, notable research in chronic bacterial prostatitis (CBP) has also been reported. Yoon et al. investigated the antibacterial effect of reduced-size catechin (nanocatechin), intending to increase its dissolution rate and bioavailability. Catechins are polyphenolic antioxidants that appear in some plants as secondary metabolites. Green tea (GT) catechins are well known to exhibit a broad range of medical uses. For example, GT catechins are valuable antimicrobial agents (Reygaert, 2018); however, their oral bioavailability is limited by its metabolism, being converted into inactive products by intestinal bacteria (Hollman et al., 1997; Clifford et al., 2013). Therefore, the authors developed GT nanocatechins (GTN) coated with hydroxypropyl methylcellulose (HPMC), included as an enteric coating to improve GTN absorption. The authors compared the antibacterial activity of GTN, in contrast with catechin and ciprofloxacin (recommended antibiotic to treat prostatitis) using an animal model. Results showed that the three formulations reduced colony-forming units (CFU) of *E. coli* significantly, in contrast with the control group. Moreover, the GTN group registered a statically significant decrease of CFU in comparison with the catechin group (Yoon et al., 2011). Although GTN did not expose better antibacterial activity than ciprofloxacin, it could be considered as an adjuvant agent for antibiotics in general. Therefore, the GTN properties can be used for medical purposes, achieving a faster and more effective patient’s recovery, by hampering the development of antibiotic resistance.

Another strategy for the enhancement of natural compounds use as UTI treatments, is improving their oral bioavailability by intravaginal delivery with nanotechnology (Talegaonkar et al., 2008). For example, Kaur et al. formulated an intravaginal nanoemulsion-based gel (NBG), composed by polyphenol 60 (P60) present in GT catechins and curcumin (CUR) as antibacterial agents against UPEC. Analyzing the *E. coli* growth curve in the presence and absence of P60+CUR-NBG, the authors showed that the aqueous solution of P60+CUR inhibited the growth at 15 hours while NBG at 5 hours (Kaur et al., 2017a). A similar investigation used P60 and cranberry (CRB) to develop an NBG for intravaginal treatment of UTIs. Similar to the previously mentioned investigation, the growth curves of *E. coli* showed that the aqueous solution of P60+CRB inhibited growth at 15 hours while NBG at 5 hours (Kaur et al., 2017b). Finally, in another investigation, P60 was combined with ciprofloxacin (CF) in a nanoemulsion. In this case, the antibacterial activity was tested against *E. coli, K. pneumoniae, P. mirabilis, C. amalonatus*, and *C. diversus*. The results showed that at 4 and 10 ng/mL, the nanoemulsion inhibited the growth of all studied strains (Atinderpal et al., 2018).

More translational studies have studied release kinetics *in vitro* and pharmacokinetic studies *in vivo* (Kaur et al., 2017a; Kaur et al., 2017b; Atinderpal et al., 2018). The importance of presenting pharmacokinetic and pharmacodynamics’ studies of NPs lies in the fact that these would allow their escalation to clinical assays, becoming closer the possibility for their clinical use. To determine *in vitro* drug release of P60 and CUR from P60+CUR-NBG Kaur et al. employed a dialysis bag immersed in simulated vaginal media. Results showed sustained release pattern for both the P60 and CUR within 12 hours. P60 achieved the maximum release (91 ± 0.16%) in 8 hours while CUR (84 ± 0.21%) in 5 hours. For the *in vivo* pharmacokinetic analysis, the P60 was radiolabeled with ^99m^Tc to prepare radiolabeled-P60+CUR-NBG and radiolabeled-aqueous solution of P60+CUR. Then, both preparations were administrated intravaginally in rats and the concentration of radioactivity was calculated in percentage per gram of the total administered dose at different hours. The concentrations of radiolabeled-P60+CUR-NBG in kidney and bladder after 3 hours of administration was 3.07 ± 0.15 and 3.35 ± 0.45 respectively, which was higher than the obtained after administering radiolabeled-aqueous solution (radioactivity of 1.66 ± 0.30 for kidney and 1.48 ± 0.20 for bladder) (Kaur et al., 2017a). In the next investigation, *in vitro* drug release of P60 and CRB from P60+CRB-NBG was performed employing simulated vaginal media with porcine vaginal mucosa. In this case P60 showed similar maximum release (90.92 ± 0.6% in 8 hours) and CRB (99.39 ± 0.5% in 6 hours) from NBG. For the *in vivo* pharmacokinetic analysis a similar procedure was carried out and the obtained results were also similar. The radioactivity of radiolabeled-P60+CRB-NBG in kidney and bladder after 3 hours of administration was 3.20 ± 0.16 and 3.64 ± 0.29 respectively, while radiolabeled-aqueous solution showed a radioactivity of 1.21 ± 0.28 in kidney and 1.88 ± 0.14 in bladder (Kaur et al., 2017b). The investigation performed by Atinderpal et al. used a dialysis bag sank in simulated vaginal media for the *in vitro* drug release analysis. Herein, P60 and CF exposed the maximum release at 7 hours (94.8 ± 0.9% and 75.1 ± 0.15%, respectively) from P60+CF nanoemulsion. In this study authors also radiolabeled P60 with ^99m^Tc and followed similar procedure for the *in vivo* pharmacokinetic analysis. The results indicated that the concentration of radioactivity of radiolabeled-P60+CF nanoemulsion after 3 hours in kidney and bladder were found to be 3.50 ± 0.26 and 3.81 ± 0.30, respectively, contrasting radiolabeled-P60+CF aqueous solution, which showed a radioactivity of 1.21 ± 0.11 in kidney and 1.34 ± 0.12 in bladder (Atinderpal et al., 2018). Results evidenced that nanoemulsions administrated *via* intravaginal could cross the vaginal mucosa efficiently, reach target organs (kidney and bladder) and combat uropathogens in UTIs in a rat model. This could be attributed to the mucoadhesive properties of nanoemulsions and their components. This highlights the importance in intravaginal administration route in the treatment of UTIs considering that the most affected population of UTIs are women. Furthermore, this strongly supports the use of natural compounds like GT catechin, CUR, and CRB for UTI treatment when administered using nanotechnology, since it allows to enhance compounds pharmacokinetics. Besides, it permits their administration by diverse routes, using different pharmaceutical forms, overcoming the oral route limitations. Nevertheless, the advantages of using natural compounds within nanosystems is not restricted to women, but also for the treatment of men prostatitis, among others.

In another example of the relevance of *in vitro* drug release studies, Fong et al. used CHX as an organic modifier (OM), as well as an active ingredient (Styan et al., 2008), by including it in a system of silicate NPs within polyurethane nanocomposites (PUNCs) for sustained release. This OM allowed the adequate dispersion of silicate NP (hydrophilic) inside the hydrophobic PUNC-polymer matrix (Styan et al., 2008). Furthermore, CHX was employed to obtain montmorillonite (MMT), a phyllosilicate mineral with a nanolayered structure (Fong et al., 2010). Then, PUNCs were functionalized with CHX-modified MMT (PUNC-CHX-MMT), additionally they loaded 1% w/w and 2% w/w of free CHX to the PUNC-CHX-MMT. Later, the authors assessed the antibacterial properties against *S. epidermidis* of PUNC-CHX-MMT in contrast with PUNC-CHX-MMT+1%CHX and PUNC-CHX-MMT+2%CHX. Results showed that PUNCs have sustained antibacterial activity in the urinary tract model achieving negative *in vitro* bladder cultures for longer periods (PUNC-CHX-MMT+2%CHX (~50 days); PUNC-CHX-MMT+1%CHX (~25 days); and PUNC-CHX-MMT from 12 to 20 days). Later, the *in vitro* released of CHX was determined as a ratio of the theoretical amount of CHX loaded into the materials, plotted against the square root of time. The data obtained fitted with the Higuchi model, suggesting a diffusion release mechanism of CHX from the PUNCs. The results also showed that CHX had an initial burst release from PUNCs, probably due to thermodynamic CHX interaction with material surface, followed by a retarded rate of drug release, thus allowing the dissolution of CHX effective antibacterial concentrations. Thus, like similar studies here discussed, these NPs showed a sustained release profile, mainly depending on polymer properties and drug loading (Fong et al., 2010).

Venkat et al. studied kanamycin-chitosan nanoparticles (KMCSNPs) against *E. coli* and *P. mirabilis*. They immobilized these NPs on the surface of a polyurethane urethral stent (PUS), aiming to prevent UTI episodes. Results revealed that KMCSNPs-PUS possessed the highest antibacterial activity compared with the PUS functionalized with blank chitosan (CS) NPs (CSNPs-PUS), probably due to the polycationic nature of KMCSNPs-PUS. Their positive charged surface interacts more efficiently with the negative charge on bacterial cell membranes, leading to its disruption. Nevertheless, it cannot be ruled out that this can also be associated to synergistic effects of kanamycin and CSNPs. Additionally; drug release studies were performed by the inversion of KMCSNPs in artificial urine. Then, the amount of KM present in the medium was quantified in different intervals of time. Results demonstrated an initial release of 10-15% of KM within 12 hours, followed by a controlled release of drug within 7 days. This sustained release significantly reduced the risk of biofilm formation in artificial urine (Venkat Kumar et al., 2016).

Both investigations showed the potential of NPs-loaded on catheter or stent materials to prevent biofilm formation and combat urophatogens. Furthermore, the *in vitro* drug release results were associated with their antimicrobial/antibiofilm activity, suggesting high clinical value.

Moreover, nanosystems have been used for the development of a potential vaccine against UPEC. In this context, Crecente et al. engineered polymeric nanocapsules (NCs) to deliver the IutA antigen. IutA is an aerobactin receptor associated to UPEC iron uptake (Tokano et al., 2008), which was included in the NC to achieve an early presentation of the antigen to the organism, enhancing antibody production. In this NCs, the antigen IutA was entrapped between CS and dextran sulfate layers. These NCs had a vitamin E oily core where the hydrophobic immunostimulants can be harbored, whilst the hydrophilic polymeric surface anchors polar molecules efficiently (*e.g.* proteins). To determine the efficacy of the vaccine, the humoral response in mice pre-immunization was assessed. Results revealed that the group treated with IutA NCs increase de IgG levels against the IutA protein 10-fold higher than the group treated with IutA adsorbed into Alum adjuvant. This highlights the potential of the NCs as a vaccine against UPEC strains (Crecente-Campo et al., 2018). This investigation provided considerable advances in the importance of using nanotechnology to develop nanovaccines and their potential for preventing UTIs and CAUTIs. The vaccine development possesses great potential for public health applications, since it could reduce the number of UTI cases each year, the antibiotics use, and the development of antibiotic resistance.

### Nanodiamonds

Nanodiamonds (ND) are carbon-based nanoparticles that have been studied as promising candidates for drug delivery. In general, carbon nanomaterials exert their antibacterial activity by direct interaction between their surface chemical groups and the bacterial wall, thereby inducing physical damage and metabolism inhibition. Interestingly, NDs enhance these antibacterial properties due to their physical and chemical characteristics such as small size, diverse and adjustable surface functionalization, chemically inert core, and their capability to be internalized into mammalian cells (Faklaris et al., 2008). Moreover, NDs are biocompatible and less cytotoxic in comparison to other carbon-based NPs, acquiring more importance in biomedicine (Zhu et al., 2012) (**Table 2**). To support this hypothesis, Beranová et al. evaluated the antibacterial effect of NDs against *E. coli*, showing that NDs exerted antibacterial activity in a concentration dependent manner (5 µg/mL inhibited 25% of bacteria growth, while 50 µg/mL or more inhibited bacteria growth completely). The authors concluded that the bactericidal effect may have been due to the clustering of NDs around bacteria, which would probably interrupt essential cellular functions (Beranová et al., 2012).

**Table 2:** Physicochemical characteristics of nanodiamonds.

Nanodiamonds	REF	Functional group	Diameter (nm)	Tested uropathogens	Activity
Nanodiamonds	(Iyer et al., 2018)	–	6	UPEC	Antibacterial
			25		
Trimeric thiomannoside cluster conjugated to nanodiamond particles	(Khanal et al., 2015)	Thiomannoside	125 ± 9	*E. Coli*	Antiadhesive

On the other hand, Iyer et al. evaluated the ability of two different sizes of NDs (6 and 25 nm NDS) for being endocytosed into human bladder cells and kill intracellular UPEC, using T24 bladder cells infected by an invasive UPEC strain. Infected cells were then treated during two hours with 200 µg/mL of NDs. The results indicated that 6 nm NDs displayed better antibacterial effects on UPEC than acid-treated 25 nm NDs. Internalization assays were performed in the same cells using transmission electronic microscopy (TEM). Microscopy images showed that acid-treated 6 and 25 nm NDs were associated and internalized into bladder cells suggesting their utility against UPEC (Iyer et al., 2018). In this research area, Khanal et al. have developed trimeric thiomannoside clusters conjugated to NDs (ND-Man3) as anti-adhesive nanoparticles. The aim of ND-Man3 was targeting *E. coli* type-1 fimbriae, its major virulence factor. The efficiency of the ND-Man3 to inhibit bacterial adhesion to cell surfaces mediated by type 1 fimbriae was evaluated with two different assays: the inhibition of yeast agglutination; and inhibition of bacterial adhesion on the T24 bladder cells. The inhibition of yeast agglutination assay evaluates the capacity of *E. coli* type 1 fimbriae to recognize mannosylated residues on yeast surface, which is visualized by their aggregation in positive samples. The results revealed ND-Man3 inhibited the adhesion of bacteria to yeast cells 91 times more than unconjugated NDs. In addition, in the case of the bacterial binding to T24 bladder cell inhibition assay, the authors tested the ability of ND-Man3 to interfere with bacterial FimH-mediated recognition of T24 cells. The results showed that ND-Man3 inhibited adherence to T24 cells 133 times more than unconjugated NDs (Khanal et al., 2015).

Considering the aforementioned, NDs intrinsic antimicrobial properties are a promising approach for UTI management, since they have showed to be effective in the elimination not only of the extracellular bacteria, but also intracellular. This characteristic is very important because urinary tract pathogens like UPEC can invade host cytosol and exponentially grow there, forming biofilm-like inclusions called intracellular bacteria communities (IBC) (Anderson et al., 2003; Conover et al., 2016). These IBC cause chronic and recurrent infections and are very difficult to eliminate due to the inability of many antibiotics to cross cell membranes (Maurin and Raoult, 2001; Anderson et al., 2003). Hence, the use of NDs as antimicrobials would overcome classic antibiotics limitations.

The presented studies show the potential of organic nanoparticles as carriers, adjuvants, or drugs in UTI treatment. They highlight their use as coating materials for different urinary tract devices (Fong et al., 2010; Beranová et al., 2012; Khanal et al., 2015; Phuengkham and Nasongkla, 2015; Francesko et al., 2016; Venkat Kumar et al., 2016; Macedo et al., 2017; Ludwig et al., 2018; Lopes et al., 2019; Srisang and Nasongkla, 2019). Thus, this kind of therapy would be destined specifically to hospitalized and catheterized patients. Among this research the studies conducted by Fong et al. and Venkat et al. standout due to their *in vitro* drug release experiments. Thereby showing the possible behavior these NPs would have inside the body when used as catheters coating to prevent and avoid CAUTIs. Furthermore, the use of intravaginal administration of nanoemulsions based on natural compounds is one of the most promising alternatives to improve the actual treatment against UTIs (Kaur et al., 2017a; Kaur et al., 2017b; Atinderpal et al., 2018). This is due to natural compounds’ benefits for human health and their biocompatibility, in addition to the inherent benefit that a local administration represents. It is important to emphasize that these studies that propose the intravaginal administration route are the only ones that analyzed *in vitro* and *in vivo* pharmacokinetics parameters making this administration route the most suitable used in the treatment of UTIs in women. Another strategy to achieve local administration of nanoparticles is the injection directly into the bladder, as suggested by Lui et al. (2015), Iyer et al. (2018) and Khanal et al. (2015). In that context, it is noteworthy that in all previous cases a local administration route is preferred for the treatment of UTIs with nanoparticles, either by intravaginal or intravesical route for both, inpatients and outpatients. Moreover, the use of nanosystems capable of inactivating or killing urinary tract pathogens as catheters coating would prevent CAUTIs and reduce the formations of biofilms. In addition, vaccination is gaining importance in the search for a novel strategy towards UTIs prevention, as shown by the results obtained by Crecente et al. using subcutaneous vaccines for the reduction of the increasing number of UTI and CAUTI and the prevention of antibiotic resistance (Crecente-Campo et al., 2018).

## Inorganic Particles

Inorganic nanocomposites are widely investigated in the literature due to a wide variety of potential applications in different fields, especially in biomedicine (Altavilla and Ciliberto, 2017). Within the biomedical research, many inorganic materials are explored for their potential benefits in the treatment of UTI. These new nanocarriers or nanoscaled materials include silver, copper, iron, gold, and others. In this section the used of these nanomaterials in UTIs is assessed.

### Silver-Based Nanoparticles

Silver is recognized as a promising strategy against microorganisms, since it disrupts both, cell wall and metabolic pathways (Sim et al., 2018). The use of silver-based nanosystems to treat UTIs has been studied isolated and in combination with other materials (**Table 3**). For example, Syed et al. assessed the antibacterial effect of silver nanoparticles (AgNps) against *E. coli* and *S. aureus* isolated from CAUTI patients. In this investigation, the bactericidal potential of AgNps was assessed by direct colony count. The number of viable bacteria was measured after the exposure of 10^5^ CFU/mL to different concentrations of AgNPs suspension (0, 10, 50, and 100 μg/mL) after 24 hours of incubation. Results showed that AgNps reduce the number of viable cells for both, *E. coli* and *S. aureus*, in a concentration dependent manner (Syed et al., 2009). On another research, El-Batal et al., developed silver-boron nanoparticles (AgB-NPs) and evaluated their antimicrobial activities against MDR urinary tract pathogens. These AgB-NPs had a MIC of 3.90 µg/ml against *E. coli*, 7.81 µg/ml against *S. aureus*, and 1.95 µg/ml against *C. albicans.* After 24 h of incubation, through the agar-disc diffusion test, the zone of inhibition revealed that AgB-NPs were effective as antimicrobials at a concentration of 25 µg/ml. The best action was displayed against *E. coli* (18.0* mm* ZOI) and *S. aureus* (16.0 mm ZOI); additionally, they exhibited effective antifungal effects, with great potency against *C. albicans* and *C. tropicalis* (20.0 mm and 15.0 mm ZOI, respectively) (El-Batal et al., 2019). Another investigation explored the development of Ag/ZnO nanoparticles alone and in combination with ciprofloxacin and assessed their efficacy against an *E. coli*-induced UTI rat model. These Ag/ZnO NPs exerted a MIC and minimum bactericidal concentration (MBC) of 32 µg/mL and 512 µg/mL, respectively. Additionally, the use of the 512 µg/mL Ag/ZnO NPs in combination with ciprofloxacin reduced the severity of renal cortical thickness of pyelonephritic rats (Khoshkbejari et al., 2015). In a different study, the authors investigated the synergism between AgNPs and antibiotics against MDR uropathogens (*E. faecium, S. aureus, A. baumannii, E. cloacae*, three different isolates of *E. coli, K. pneumoniae, M. morganii* and *P. aeruginosa*). Ampicillin (AMP) and amikacin (AMK) were assessed alone and in combinations with AgNPs against the MDR clinical strains isolated from CAUTI patients. All clinical isolates showed a MIC between 4-16 µg/mL and 4–128 µg/mL to AgNPs and amikacin, respectively, while all Gram-negative strains resulted to be ampicillin resistant. The combinations of AgNPs + AMK showed a synergistic effect reducing the MIC by 2 to 32-fold. On the other hand, AgNps + AMP reduced *S. aureus* and *E. cloacae* MICs by 1- and 4-fold respectively (Lopez-Carrizales et al., 2018). In another research looking into optimizing the antibacterial and antibiofilm properties of AgNPs, Bhargava et al. developed L-fucose-functionalized nanoparticles (FNPs). The aim was to increase FNPs interactions with *P. aeruginosa* PAO1 using LecB lectins present on the bacteria. They compared them to similar size and concentration of citrate capped silver nanoparticles (CNPs). The authors observed that, by measuring the MBC in static conditions, only 40 µg (Ag)/mL in FNPs aliquots were required to kill all bacteria in comparison with CNPs, which required 70 µg (Ag)/mL (Bhargava et al., 2018),. In another attempt to synergistically increase the therapeutic efficacy of silver nanoclusters (NCls), branched polyethylenimine (bPEI) and silver were combined in the synthesis of bPEI-coated blue fluorescent cationic silver nanoclusters (bPEI−Ag-NCls). bPEI is an effective transfection reagent widely investigated as a non-viral vector for DNA delivery (Kunath et al., 2003). The antibacterial ability of these NCls was evaluated against twelve MDR uropathogenic strains by determining MIC through the broth dilution method. AgNO3 and PEI were used as controls. The results showed that bPEI-Ag NCls had a 2-to 3-fold lower MIC than AgNO3 and 10- to 15-fold lower MIC than that PEI. In addition, in hemolysis assays and fibroblasts cultures, it was additionally shown that these bPEI-Ag NCls had a selective cytotoxicity against bacteria, and were biocompatible with human fibroblasts and red blood cells (Huma et al., 2018).

**Table 3:** Physicochemical characteristic of silver-based NPs.

Silver based inorganic NPs	REF	Abbreviation	Diameter (nm)	ζ - potential (mV)	Tested Uropathogens	Activity
Silver nanoparticles	(Syed et al., 2009)	AgNps	9 – 24	NA	*E. coli* and *S. aureus*	Antibacterial
Silver boron nanoparticles	(El-Batal et al., 2019)	AgB NPs	25.46	NA	*E. coli, S. aureus* and *C. albicans*	Antibiofilm
Ag/ZnO nanoparticles	(Khoshkbejari et al., 2015)	Ag/ZnO NPs	12	NA	*E. coli*	Antibacterial
Silver nanoparticles-ampicilin	(Lopez-Carrizales et al., 2018)	AgNPs-AMP	4.01 ± 0.80	51.00 ± 20.20	*E. faecium, S. aureus, A. baumannii, E. cloacae, three different isolates of E. coli, K. pneumoniae, M. morganii* and *P. aeruginosa*	Antibacterial
Silver nanoparticles-amikacin	(Lopez-Carrizales et pal., 2018)	AgNPs-AMK	6.03 ± 0.87	-21.10 ± 4.63	*E. faecium, S. aureus, A. baumannii, E. cloacae, three different isolates of E. coli, K. pneumoniae, M. morganii* and *P. aeruginosa*	Antibacterial
Silver nanoparticles functionalized with L-fucose	(Bhargava et al., 2018)	FNPs	8 – 10	-17.7	*P. aeruginosa*	Antibacterial
Cationic silver nanoclusters coated with low molecular weight polyehylenimine	(Huma et al., 2018)	bPEI-Ag NCs	3	30	*E. coli, P. aeruginosa*, MRSA, *E. cloacae, E. faecalis*	Antibacterial

Silver was widely used as antibacterial agent in the 19^th^ century, until the discovery of the new antibiotics in the early 20^th^ century (Möhler et al., 2018; Sim et al., 2018). However, bacteria have progressively developed different mechanism to evade antibiotic effects (Möhler et al., 2018; Sim et al., 2018). Nowadays, the fight against infections like UTI caused by MDR pathogens represents a challenge. So, to overcome antibiotics resistance, silver is being reconsidered as antimicrobial agent. But now, the use of silver is studied in a more refined manner using strategies such as nanotechnology. AgNPs are already being used for catheter coating to prevent CAUTI due to their antimicrobial and antibiofilm potential. However, their ability to be combined or functionalized increases the interest in the use silver-based NPs to develop powerful molecules for UTI treatment, one of the most MDR pathogens-associated infections.

#### Green Silver-Based Nanoparticles

Inorganic NPs can be engineered by physicochemical and biological pathways. Physicochemical methods to synthetize NPs employ strong reducing agents, and organic solvents (Pal et al., 2019). The use of these chemical reagents represents toxicity and environmental issues. For this reason, biological synthesis methods are preferred (Iravani, 2014; Pal et al., 2019). On the other hand, biological synthesis methods comprise the use of plants, bacteria, fungi, or algae for the bioreduction of metal ions in the productions of NPs. Therefore, NPs generated by biological synthesis are free of toxic chemicals and biocompatible. For this reason, biological synthesis is considered as green synthesis, and it has also demonstrated to be simple and cost-effective (Iravani, 2014; Pal et al., 2019).

Due to the remarkable advantages of green synthesis, it is also considered in the development of new compounds for UTI treatment (**Table 4**). For example, leaf extract of *Azadirachta indica* has been used to synthesize Ag-embedded mesoporous silica nanoparticles (mSiO2-AgNPs) using the plant as reductor agent. The antifungal activity of mSiO2-AgNPs were tested against MDR *C. albicans* using various concentrations (0, 2, 8 µg/mL). The results of agar disc diffusion assays revealed thar the antifungal activity was significantly enhanced in a dose dependent manner, exposing the best activity at 8 µg/mL (Qasim et al., 2015). Likewise, another investigation used bacteria for the metal reduction (Divya et al., 2019). They used bacteria isolated from healthy coral samples (MGL-D10) to synthesize green silver nanoparticles (AgNP MGL-D10). These nanoparticles were assessed against UTI causing pathogens such as *Bacillus sp, C. albicans, E. coli, K. pneumoniae, P. aeruginosa*, and *S. aureus* by using MIC determination. Results showed that 10 µg of AgNPs were effective inhibiting a 50% of the tested microorganisms, whereas 30 µg of AgNPs showed an inhibition of 100% for pathogen growth, such as *Bacillus sp*, *S. aureus*, and *C. albicans*. In addition, *E. coli, K*. *pneumoniae*, and *P. aeruginosa* were completely inhibited using 40 µg/ml of AgNP MGL-D10. Moreover, the concentration of 50 µg/ml destroyed biofilm effectively as well (Divya et al., 2019).

**Table 4:** Physicochemical properties of green silver-based NPs.

Green silver based NPs	REF	Functional group	Diameter (nm)	ζ - potential (mV)	Tested uropathogens	Activity
Silver nanoparticles	(Qasim et al., 2015)	Hydroxyl	30-50	–	*Bacillus sp, C. albicans, E. coli, K. pneumoniae, P. aeruginosa* and *S. aureus*	Antibacterial
Ag-embedded mesoporous silica nanoparticles	(Divya et al., 2019)	–	15 - 50 AgNps/400 mSiO2	–	C. albicans	Antifungal
Silver nanoparticles	(Mishra et al., 2018)	Phenolic compounds	<100	–	*S. aureus, E. faecalis, A. baumannii, Citrobacter freundii, Enterobacter aerogenes, E. coli, K. oxytoca, K. pneumoniae, P. mirabilis, P. vulgaris*, and *P. aeruginosa*	Antibacterial
Silver nanoparticles	(Shafreen et al., 2017)	*N. palea aminoacids* and proteins	70–90	–	*E. coli*	Antibiofilm
*Piper betle* based silver nanoparticles	(Srinivasan et al., 2018)	Amide	156.4	− 23.1	*S. marcescens* and *P. mirabilis*	Antibiofilm
						Antiquorum sensing
Silver nanoparticles	(Meenakumari et al., 2000)	–	87	–	*P. aeruginosa, K. pneumoniae, E. faecalis, S. aureus* and coagulase negative *Staphylococcus*	Antibacterial

In another investigation, AgNPs were synthesized using *Anogeissus acuminata* as potential treatment of MDR urinary tract infecting bacteria. *Anogeissus acuminata* was selected due to its secondary phytochemicals with antimicrobial ethnomedical uses (Singh et al., 2016), which was expected to convey its antibacterial properties in the synthesis of AgNPs. Antibacterial effects of different concentrations of these AgNPs (5, 10, 15 µg/mL) were determined using the agar well diffusion method against eleven MDR pathogens: two Gram-positive (*S. aureus* and *Enterococcus faecalis*) and nine Gram-negative (*A. baumannii, Citrobacter freundii, Enterobacter aerogenes, E. coli, K. oxytoca, K. pneumoniae, P. mirabilis, P. vulgaris*, and *P. aeruginosa*) isolated from UTI patients. The results revealed that these green AgNPs had great concentration-dependent antibacterial activity, obtaining the largest ZOI (19 to 13 mm) at 15 µg/mL (Mishra and Padhy, 2018). In a different investigation Shafreen et al. used *Nitzschia palea* (*microalgae)* for the synthesis of green AgNPs. *Nitzschia palea* is a diatom considered as an antibiofilm agent against UPEC (Shafreen et al., 2017). These green AgNPs were developed with the objective of halt curli-*E. coli* biofilm formation. Curli are thin proteinaceous fimbriae, which mediate cell-cell interaction promoting biofilm formation (Kikuchi et al., 2005). The authors determined for these green AgNPs, a 300 ng/mL biofilm inhibitory concentration (BIC, concentration able to inhibit more than 50% of biofilm) (Shafreen et al., 2017). To determine the presence of curli-*E. coli*, it was used the analysis of specific morphological characteristics (cultures should have red, dry, and rough (RDAR) morphology). In addition, cultures ability to bind to congo red agar, as a maker of curli formation (Shafreen et al., 2017) was also measured. Results showed that when *E. coli* grew on AgNPs plates, cultures appeared with pale, smooth and wrinkled morphology, suggesting that curli-mediated biofilm formation was inhibited. Furthermore, the reduced binding to congo red dye validated the morphological results (Shafreen et al., 2017). In another investigation Srinivasan et al. used *Piper betle* leaves extract to synthetize *Piper betle*-based silver nanoparticles (PbAgNPs), since it has antibacterial activity reported (Prabha et al., 2014). In this work the authors wanted to evaluate the potential of PbAgNPs as anti-quorum sensing (QS) agent. Given that QS is related to a wide number of biological functions, including biofilm formation (Parsek and Greenberg, 2005). The anti-QS activity of PbAgNPs was evaluated by their potential to inhibit QS-mediated virulence factors such as prodigiosin and protease production, as well as biofilm formation. PbAgNPs exhibited a 16 µg/mL and 32 µg/mL MIC, against *S. marcescens* and *P. mirabilis*, respectively; whilst MIC concentrations were of 6 µg/mL for *S. marcescens* and 10 µg/mL for *P. mirabilis*. In addition, PbAgNPs exerted inhibitory effect over *S. marcescens* virulence factors reducing: i) 62-72% of prodigiosin pigment production; ii) 31-42% of protease production; and iii) 63-71% of reduction of biofilm formation. On the other hand, the results for *P. mirabilis* were significant only in biofilm inhibition (53-69%). Consequently, authors suggested that PbAgNPs had anti-QS potencial, especially inhibiting biofilm formation (Srinivasan et al., 2018). Green synthesis of AgNPs was also carried out employing two Indian medicinal plants, *Aegle marmelos* (Mujeeb et al., 2014) and *Saraca asoca* (Singh et al., 2015). The antibacterial activity of the resulting AgNPs was assessed against *P. aeruginosa, K. pneumoniae, E. faecalis, S aureus* and Coagulase Negative *Staphylococci* by the agar dilution method. Very effective antibacterial properties were found when 5 µg/mL AgNPs was assayed against all pathogens (Meenakumari et al., 2000).

### Copper-Based Nanoparticles

Because of its biocide nature and its antibacterial ability reported since Egyptian medical texts (Nunn, 2002), copper (Cu) is another common metal widely used for the synthesis of nanoparticles as nanoantibiotics, having the advantage of its lower cost in comparison with silver (Rane et al., 2018). The utility of Cu against uropathogens is principally as antibiofilm agent to prevent CAUTI (**Table 5**). To amplify this concept Shalom et al. coated urinary silicone catheters with Zn-doped CuO nanoparticles. Subsequently, their antibiofilm activity was evaluated against *E. coli*, *S. aureus*, and *P. mirabilis*, observing a biofilm persistence of 9, 8% and 0.5%, respectively. Finally, the authors performed experiments using rabbits as an *in vivo* model to which coated and uncoated catheters were applied. Rabbits catheterized with uncoated catheters developed CAUTI at day 4 of the experiment. In contrast, CAUTI was not diagnosed in rabbits catheterized with coated catheters during the time that the experiment was performed (7 days) (Shalom et al., 2017).

**Table 5:** Physical characteristics of copper-based NPs.

Copper-based NPs	REF	Diameter (nm)	Tested uropathogens	Activity
Copper oxide nanoparticles	(Shalom et al., 2017)	25 - 30	MRSA and *E. coli*	Antibiofilm
Zn-doped copper oxide nanoparticles	(Agarwala et al., 2014)	35 - 95	*E. coli, Saureus, P. mirabulis*	Antibiofilm

In another study, researchers evaluated the antibiofilm activity of commercial CuO NPs against biofilm formed from methicillin-resistant *Staphylococcus aureus* (MRSA) and *E. coli*. First, Agarwale et al., determined a 30 μg/mL and 35 μg/mL CuO NPs-MIC, for MRSA and for *E. coli*, respectively. Next, they assessed the antibiofilm activity of CuO NPs employing sub-MIC concentrations. Results indicated that CuO NPs completely inhibited biofilm formation at a concentration from 2MIC to ½MIC, while lower concentrations did not inhibit the biofilm completely (Agarwala et al., 2014).

The aforementioned exposed that Cu used into NPs reduces biofilm formation efficiently, which is associated to the damage that Cu produces in microorganisms, since it destroys microorganism’s envelope and nucleic acids leading to cell death (Rane et al., 2018). These Cu properties added to nanotechnology strategies, present Cu-based NPs as excellent candidates for their inclusion in CAUTI prevention treatments. Furthermore, the inclusion of NPs onto catheters surface reduces biodistribution and bioavailability problems, thus representing a big advantage for their future biomedical approval and commercialization.

#### Green Copper-Based Nanoparticles

Nowadays, copper nanoparticles can also be synthesized by green methods (**Table 6**). For example, Rajivgandhi et al. developed green CuO NPs using leaf extract of *Camilla japonica* as reductor agent. These NP were evaluated against beta-lactamase (ESBL) positive Gram-negative uropathogens (*P. aeruginosa* and *K. pneumoniae*). The results of disc diffusion assays revealed that the CuO NPs inhibited the growth of tested bacteria at a concentration of 100 µg/mL. In addition, reduced cell viability and loss of the cell membrane integrity was observed by laser scanning microscope in both uropathogens, supporting the evidence for Cu NPs as potentials antibacterial agents (Rajivgandhi et al., 2019). In a different research, the culture supernatant of *Serratia nematodiphila* was employed for the synthesis of green copper sulfide nanoparticles (CuS NPs), by the reduction of copper sulfate to copper sulfide (Malarkodi and Rajeshkumar, 2017). The antibiotic activity of these NPs was assessed by ZOI microbiological assay and compared with commercial antibiotics such as amikacin, amoxicillin, azithromycin, cefixime, ciprofloxacin, chloramphenicol, penicillin, nitrofurantoin, ofloxacin among others. For the ZOI determination, 120 μg/mL of CuS NPs and 30 mg of each commercial antibiotic were employed. ZOI results revealed that *E. coli* was the most sensitive bacteria to of CuS NPs, with a ZOI of 24 mm, followed by S*. aureus* (22 mm), *P. vulgaris* (17 mm) and *K. pneumoniae* (16 mm). These results were superior than those obtained with commercial antibiotics, since none of these exhibited a ZOI greater than 20mm, evidencing the high potential of CuS NPs (Malarkodi and Rajeshkumar, 2017). *Tabernaemontana divaricate* leaves extract was also used in the synthesis of CuO NPs; the plant was chosen due to its reported medical applications (antiparasitic, antibacterial, antifungal, and anti-inflammatory) due to its high content of phytochemicals (Pratchayasakul et al., 2008). Antimicrobial activity of these CuO NPs were assessed by a modified Kirby Bauer disc diffusion, against UPEC. The results exhibited that green CuO NPs presented a ZOI of 17 mm at a concentration of 25 µg/mL, in comparison to the 50 µg/mL needed of a tetracycline solution, to achieve the same ZOI. These results supported the antimicrobial potential of the synthesized green CuO NPs (Sivaraj et al., 2014).

**Table 6:** Physical characteristics of green copper-based NPs.

Green copper-based NPs	REF	Functional group	Diameter (nm)	Tested uropathogens	Activity
Copper oxide nanoparticles	(Rajivgandhi et al., 2019)	Phenolic compounds (*C. japonica*)	15 - 25	*P. aeruginosa* and *K. pneumoniae*	Antibacterial
Copper sulfide nanoparticles	(Malarkodi et al., 2017)	Hydroxyl and phenolic compounds	10–80	*K. pneumoniae, E. coli, S. aureus* and *P. vulgaris*	Antibacterial
Copper oxide nanoparticles	(Sivaraj et al., 2014)	Hydroxyl and phenolic compounds	48	UPEC	Antibacterial

### Zinc-Based Nanoparticles

Other metal with antibacterial properties used for NPs synthesis is zinc, which has potential to achieve more effective UTI treatments (**Table 7**). Zinc oxide nanoparticles (ZnO NPs) have been developed by two different methods (green and conventional chemical syntheses). The antibacterial ability of these NPs was evaluated against carbapenem-resistant RS307 strain of *Acinetobacter baumannii*, an opportunistic pathogen of urinary tract. Growth kinetics assay showed that the growth of *A. baumannii* were inhibited by chemically ZnO NPs more than by green synthesized ZnONPs. This effect was attributed to the ROS production by the chemically synthesized NPs (Tiwari et al., 2018). In other research, authors investigated the capacity of ZnO NPs to reduce both biofilm formation and antigen 43 (Ag43) expressions in UPEC CFT073. Ag43 is an important surface adhesin encoded by the *E. coli*-*flu gene* (Zalewska-Pia Tek et al., 2015). *E. coli* surface also presents the Ag43 receptor, which mediates bacteria-bacteria adherence, favoring their auto-aggregation, essential for the biofilm formation (Ulett et al., 2007). The ZnO NPs MIC was determined against UPEC isolated from urine samples of inpatients and outpatients, using the agar diffusion methods and different NPs concentrations. Some isolated bacteria resulted to be ESBL-positive, thus resistant to third generation cephalosporins. The MIC obtained for no ESBL-producing *E. coli* was 2971 µg/mL, while for ESBL-producing *E. coli* the MIC was 3541 µg/ml. With the MIC concentration, they measured the antibiofilm activity of the ZnO NPs by microtiter plate assay. For that, they first measured the extend of biofilm by optical density (OD_490_), considering values of ˂0.1, 0.1-0.2, 0.2-0.3, and ˃0.3, representing non, weak, moderate, and strong biofilm former respectively (Naves et al., 2008). They determined that the biofilm formation was fully inhibited in about 20% of isolates with strong biofilm formation, 14% in moderates, and 16% in weaks. Finally, they assessed the level of the *flu gene* expression by Real-Time PCR assay, determining that ½MIC concentration of NPs significantly reduced the *flu gene* expression in these bacteria (Shakerimoghaddam et al., 2017). Hence, they found a direct correlation between biofilm strength and *flu gene* expression. Other authors have developed Magnesium-doped zinc oxide nanoparticles (ZnO : MgO NPs), and evaluated their antibiofilm activity against CAUTI*-*isolated *P. mirabilis*. To evaluate the effect of ZnO : MgO NPs over *P. mirabilis* biofilm, they covered glass coverslips with polymer and nanoparticle dispersion containing 1% of HPMC and different percentages of ZnO : MgO NPs (0.0004, 0.0011, 0.0023 and 0.0053% w/w). They used glass coverslip with and without 1% HPMC as controls. Then, coated coverslips were incubated with *P. mirabilis* culture for 7 days. Coverslips were then subjected to immunofluorescence staining and confocal microscopy image acquisition. Deep image analysis and mathematical morpho-topological descriptors (bacterial volume, and number, and extracellular volume) revealed that each NPs concentration induced a different biofilm behavior. The best concentration of these NPs to inhibit biofilm formation was 0.0011% w/w, maintaining the smallest morpho-topological parameters values during all assays compared to controls and the other NPs concentrations (Iribarnegaray et al., 2019). In a different study, Hosseine et al. evaluated the ZnO NPs effect on agglutinin-like sequence (*ALS*) 1 and *ALS*3 gene expression. ALS are proteins present in cells surface of *C. albicans* with a key role in biofilm formation, especially ALS3 (Nailis et al., 2010). The antifungal effect of ZnO NPs was carried out by MIC determination against patients*-*isolated *C. albicans* strains. The results of MIC determinations showed that all strains were sensitive to ZnO NPs at a concentration of 17.76 µg/mL. Then authors analyzed the effect of ZnO NPs on the expression of ALS genes by Real-Time PCR. For that, they treated *C. albicans* strains with a sub-MIC concentration of ZnO NPs. Results indicated that, after treatment with the sub-MIC concentration of ZnO NPs, the expression of ALS1 and ALS3 decreased significantly, suggesting ZnO NPs as an effective antibiofilm agent (Hosseini et al., 2019). In another investigation, Hosseini et al. studied the influence of ZnO NPs on *C. albicans* biofilm derived from urinary-catheters isolated strains, some of which were fluconazole resistant. First, the authors determined the MIC of ZnO NPs for all strains obtaining a MIC of 28 µg/mL for fluconazole-susceptible strains and of 47 µg/mL for fluconazole-resistant. Later, the antibiofilm assay showed that concentrations of 29 µg/mL fully inhibited biofilm formation in 80% of susceptible strains and 50 µg/mL inhibited biofilm formation in 100% of resistant strains (Hosseini et al., 2018). On another research, Bhande et al. assessed the synergism between ZnO NPs and beta-lactam antimicrobials (cefotaxime, ampicillin, ceftriaxone and cefepime) against a panel of clinically isolated UTIs ESBL producers (*E. coli, P. aeruginosa, S. paucimobilis*, and *K. pneumoniae*). The MIC determination was 80 µg/ml for *E. coli*, 60 µg/ml for *K. pneumoniae*, 30 µg/ml for *P. aeruginosa*, and 50 µg/ml for *S. paucimobilis*. In addition, time-kill assay by synergistic compound (ZnO NPs + beta-lactam antibiotics) was monitored using broth dilution and agar diffusion method (% fold inhibition) for ESBL producers. Results exposed that both, antibiotics and ZnO NPs delayed the normal pathogens exponential growth. Nevertheless, when they were treated with the different combinations of ZnO NPs + beta-lactam antibiotics, it was observed a sudden impairment in the exponential phase, and a very low stationary phase during the growth transition (Bhande et al., 2013).

**Table 7:** Physicochemical characteristics of zinc-based NPs.

Zinc-based NPs	REF	Functional group	Diameter (nm)	ζ - potential (mV)	Tested uropathogens	Activity
Zinc oxide nanoparticles	(Tiwari et al., 2018)	Carboxylate	29.79	–	*Carbapenem resistant Acinetobacter baumannii*	Antibacterial
Zinc oxide nanoparticles	(Shakerimoghaddam et al., 2017)	NA	10–30	–	UPEC	Antibiofilm
Magnesium-doped zinc oxide nanoparticles	(Iribarnegaray et al., 2019)	Hydroxyl/Hydroxypropyl methylcellulose	15.2	-1.9	*P. mirabilis*	Antibiofilm
Zinc oxide nanoparticles	(Hosseini et al., 2019)	–	20 - 40	–	*C. albicans*	Antifungal
Zinc oxide nanoparticles	(Hosseini et al., 2018)	–	20 - 50	–	*C. albicans*	Antibiofilm
Zinc oxide nanoparticles/cefotaxime/ampicilin/ceftraxone/cefepime	(Bhande et al., 2013)	–	15	–	*E. coi, P. aeruginosa, S. paucinomobilis* and *K. pneumoniae*	Antibiofilm
Green Zinc oxide nanosheets	(Rajivgandhi et al., 2018)	Hydroxyl	500	–	*P. mirabilis* and *E. coli*	Antibiofilm

#### Green Zinc-Based Nanoparticles

Green synthesis has also been employed for the development of zinc-based NPs. Herein the authors engineered ZnO nanosheets (NSs) to be applied in UTIs treatment. They used the actinomycete *Nocardiopsis* sp. GRG1 (KT235640) to synthesized green ZnO NSs, for the treatment of MDR uropathogens. The antibacterial activity was carried out through Kirby bauer disc diffusion assay. Results indicated that 25 µg/mL of ZnO NSs gave the maximum ZOI against *P. mirabilis* (27* mm*)   and 24 mm against *E. coli*, whereas extract only exhibited a ZOI of 10 and 5 mm for *P. mirabilis* and *E. coli*, respectively. In addition, the antibiofilm properties were evaluated using 20 µg/mL of ZnO NSs. After 24 hours of treatment with ZnO NSs, it was observed an biofilm inhibition in about 92% and 90% for *P. mirabilis* and *E. coli* about, respectively (Rajivgandhi et al., 2018). These results support the utility of ZnO NSs for the treatment of UTI MDR pathogens.

### Gold-Based Nanoparticles

Gold-based NPs (AuNPs) (**Table 8**) have shown potent bactericidal activities by different mechanisms, including membrane damage, protein inactivation and DNA replication inhibition (Cui et al., 2012). For that matter, the antibacterial activity of functionalized AuNPs has been evaluated, using a wide range of different gold-cationic characteristics, such as chain length, non-aromatic, and aromatic properties, for those purposes. The antibacterial activity was first tested on a *E. coli* laboratory strain (DH5a); using the broth dilution method, the researchers determined that all functionalized AuNPs inhibited *E. coli* proliferation at nanomolar concentrations. Next, they tested the antibacterial ability of the most potent NPs against *E. coli* growth. These specific AuNPs carried an n-decan end group (NP3) and inhibited *E. coli* proliferation at 32 nM (Li et al., 2014). In another study, the authors conjugated AuNPs with chlorhexidine (Au-CHX NPs), where they evaluated the antibacterial and antibiofilm activity against twenty strains of *K. pneumoniae* isolated from UTI patients and one reference strain (ATCC 13882). The antibacterial effects were investigated by the broth dilution method, while the antibiofilm activity was evaluated by the microtiter plate method. Results revealed that the MIC necessary to inhibit *K. pneumoniae* ATCC 13882 strain was 25 µM, whereas for all the clinical isolates it was 100-200 µM. Regarding the ability for interrupting the biofilm formation, 25 µM reduced the *K. pneumoniae* ATCC 13882 strain biofilm to almost 85%, whilst 100 µM were required to reduce the biofilm of clinical isolates to about 90%. It was also determined that concentrations of 100 µM of Au-CHX NPs were able to disrupt the preformed biofilm of all isolates (Ahmed et al., 2016). A different investigation with AuNPs developed silica coated gold nanorods (AuNRs-SiO2) loaded with verteporfin (VP). VP is a photosensitizer, clinically approved as efficient near infrared (NIR) nanostructures for PDT (Turcheniuk et al., 2015). Herein the aim was to use a continuous wave (CW) or a pulsed-mode laser to eliminate *E. coli* related with UTI. To determine the potential of the AuNR-SiO2–VP as antibacterial photodynamic probe, their bactericidal capacity was assessed under pulsed laser illumination. Using this laser at 710 nm (1 W/cm^2^), repeated irradiations of AuNR-SiO2–VP, AuNR-SiO2 and VP were applied on *E. coli* strains. The results showed that *E. coli* UTI89 was not inactivated at 4 mM neither with AuNR-SiO2 nor VP, while AuNR-SiO2–VP eradicated an infectious dose of 10^4^ CFU/mL of *E. coli* (Turcheniuk et al., 2015).

**Table 8:** Physicochemical characteristics of gold-based NPs.

Gold-based NPs	REF	Functional group	Diameter (nm)	ζ - potential (mV)	Tested Uropathogens	Activity
Gold nanoparticles	(Li et al., 2014)	Amino-pyrimidine	3.1	–	*E. coli*	Antibacterial
Gold nanoparticles	(Li et al., 2014)	Quaternary ammonium	2	–	*E. coli*	Antibacterial
Gold nanoparticles conjugated with chlorhexidine	(Ahmed et al., 2016)	–	20 - 100	–	*K. pneumoniae*	Antibiofilm
Silica coated gold nanorods loaded with verteporfin	(Turcheniuk et al., 2015)	–	20 ± 3 nm (length/diameter AR 3.5 ± 0.3)	-16	*E. coli*	Antibacterial

Thus, gold nanosystems have great potential for UTI treatment, since they allow the incorporation of other compounds, potentiating their activity using different strategies for bacteria eradication.

### Silica-Based Nanoparticles

Another inorganic molecule used to improve actual UTI treatments is silica, which has been generally used in combination with other materials (**Table 9**). For example, Tameemi et al. synthesized nanostructures of silica-titanium sieves (Si-Ti-Sv) as nanocarriers for izohidrafural, a new antibacterial agent, obtaining Izo3-Si-Ti-Sv. The antibacterial activity of these nanocarriers was assessed against different bacteria strains of nosocomial UTI pathogens (*E. coli*, *K. pneumoniae*, *P. mirabilis*, *M. morganii*, and *E. faecalis*). The results showed Izo3-Si-Ti-Sv had greater antibacterial potential than Si-Ti-Sv and nitrofurantoin. Furthermore, Si-Ti-Sv exposed higher antibacterial activity than nitrofurantoin but lower than Izo3. The authors attributed this synergistic effect to the photolytic properties of titanium dioxide, in the form of anatase, which is present in the hybrid Si-Ti-Sv and generates ROS, thus destroying cells. The increased antimicrobial activity of Izo3, in regard to nitrofurantoin was explained by the isoniazid moiety, a powerful antimicrobial present in the molecular structure of the drug (Al Tameemi et al., 2017). In a different research, the authors conjugated SiO_2_ NPS with tannase, an enzyme that catalyzes the hydrolysis of tannic acid ester bonds to produce gallic acid and glucose (Singh et al., 2019). Tannase was obtained and purified from *Serratia marcescens*. The conjugation was done by feeding and pulse methods (Nsayef Muslim et al., 2017). Then they compare the antibacterial activity of SIO_2_ against UTI-related pathogens (*S. agalactiae*, *S. aureus, K. pneumonia, E. coli, P. aeruginosa, E. aerogenes* and *S. marcescens*) with ciprofloxacin, SiO_2_ NPs alone, and partially purified tannase. The best results against tested pathogens were achieved with tannase-SiO2 NPs conjugated feeding method (Nsayef Muslim et al., 2017). Mesoporous silica nanoparticles (MSNPs) have also been investigated as part of possible future UTIs treatment because their physical and chemical characteristics are easily modifiable. The ability to alter at will characteristics such as pore size, surface area, stability to organic solvents, biocompatibility, and others, facilitates the incorporation of organic and inorganic particles, thus enhancing antibacterial properties (Stein et al., 2000). Based on this information, mesoporous silica nanoparticles were functionalized with phenazine-1-carboxamide (PCN) (PCN-MSNPs) and used as antimicrobial coating on silicone urethral catheters. Microbial phenazines are nitrogenous aromatic compounds reported to exhibit interesting bioactivities including antimicrobial properties (Shanmugaiah et al., 2010). In this research, the authors evaluated the antifungal activity of PCN-MSNPs against different strains of *C. albicans* by Minimum Fungicidal Concentration (MFC) assay, in Muller-Hinton Broth (MHB). In this research, it was shown that PCN-MSNPs exhibited a MFC of 7.8-15.6 µg/mL (two-fold lower than PCN) against *C. albicans*, similar to the standard MZ. The authors also did a polymicrobial inhibition assay using *agar well diffusion* method against a mixture of *C. albicans* and *S. aureus.* The assay revealed that both PCN and PCN-MSNPs inhibited the growth of mixed pathogens, but PCN-MSNPs showed a greater inhibitory effect, which was stable up to 120 hours. Finally, antibiofilm activity of PCN and PCN-MSNPS was assessed against *C. albicans* and the mixture of *C. albicans* and *S. aureus*, in microtiter plates. The results of *C. albicans* biofilm inhibition assay showed that PCN inhibited the biofilm at a concentration ranging from 80–95 μM, whereas, PCN-MSNPs needed only a dose ranging between 34 to 44 μM. Similar results were found on mixture of pathogens biofilm, where PCN-MSNPs inhibited biofilm formation at a concentration of 53 μM, while PCN showed biofilm inhibition at 105 μM (Kanugala et al., 2019).

**Table 9:** Physicochemical characteristics of silica-based NPs.

Silica-based NPs	REF	Functional group	Diameter (nm)	Tested uropathogens	Activity
Silica titania sieves containing izohidrafural	(Al Tameemi et al., 2017)	Hydroxyl	Size of pores 4.5 nm	*E. coli, K. pneumoniae, Morganella morganii, P. mirabilis* and *E. faecalis*	Antibacterial
Siliconoxide nanoparticles conjugated to tannase	(Nsayef Muslim et al., 2017)	Tannase	92	*S. agalactiae, S. aureus, K. pneumonia, E. coli, P. aeruginosa, E. aerogenes* and *S. marcescens*	Antibacterial
Phenazine-1-carboxamide functionalized mesoporoussilica nanoparticles	(Kanugala et al., 2019)	Phenazine 1 carboxamide	Size of pores 6.43 nm	*C. albicans*	Antifungal
				Mixture of *C. albicans* and *S. aureus*	Antibacterial
				*C. albicans* and *S. aureus*	Antibiofilm

### Other Inorganic Nanoparticles

A different group of inorganic materials has been employed in the development of nanoparticles to enhance UTI treatments (**Table 10**). Within this group, hydroxyapatite nanoparticles (nano HA) have been used to develop an antimicrobial coating for urethral catheters with the aim to prevent biofilm formation and CAUTI. *In vivo* studies in rabbits were conducted with a control catheterized with standard silicon-latex catheters, while the study group received nano-HA coated catheters. To evaluate the biofilm formation on catheters, 1 cm of the distal segment of each catheter was plunged in a transferable culture media and analyzed by scanning electron microscopic (SEM) under 15 kV to determine biofilm thickness. The most frequent bacteria recovered in urine and on the catheter surface in the control group were *E. coli, Staphylococcus species, P. mirabilis* and *E. cloacae*. The results showed that at the end of seven days of the catheterization period, the biofilm formation over catheters surface was significantly thinner in the study group, regarding the control group. After the 7 days, the urethra and bladder of each rabbit were excised and analyzed with hematoxylin-eosin. The results indicated that nano-HA coated urethral catheters did not produce histological adverse changes or particle penetration in the urothelium (Evliyaoğlu et al., 2011). In another study, sulfur nanoparticles (SNPs) were green synthesized with *Catharanthus roseus* leaves extract as reductor agent. The antibacterial efficacy of these SNPs was single evaluated and in combination with antibiotics (amoxicillin, chloramphenicol, ciprofloxacin, gentamicin, imipenem, kanamycin, neomycin, norfloxacin, ofloxacin, trimethoprim) against MDR uropathogens, using disc diffusion method against uropathogenic bacteria isolated from 351 human urine samples. The isolated pathogens were *E. coli, P. mirabilis, P. aeruginosa, K. pneumoniae, E. faecalis* and *S. aureus*. The results showed that SNPs alone or combined with antibiotics had inhibition activity against all assessed pathogens, being 14.66 mm the maximum ZOI observed for SNPs-Amoxicillin combination against *E. coli* (Paralikar et al., 2019). In yet another example, tungsten nanoparticles (WNPs) were assessed against biofilm formation from CAUTI patients isolated *E. coli* strains. Antibacterial properties were assessed against these bacteria and against (*S. aureus*) reference strain (ATCC 6538). To measure the antibacterial potential of these novel WNPs authors applied MIC determination and the results revealed that while 6000 µg/mL of cefotaxime were necessary to inhibit *E. coli* growth and 2000 µg/mL were needed to avoid *S. aureus* growth, only 1500 µg/mL of WNPs were enough to avoid *E. coli* and *S. aureus* growth (Syed et al., 2010). Finally, nickel oxide nanoparticles (NiO NPs) are one of the most promising metal oxides used for biomedicine and drug delivery applications (Bano et al., 2016; Kganyago et al., 2018). Pure NiO NPs and Nd^3+^ doped NiO NPs (Nd-NiO NPs) antibacterial have been tested, in comparison to commercial antibiotic treatment (erythromycin). The antibacterial ability of NPs was measured by the well diffusion method against *P. mirabilis*. Results indicated that NiO and Nd-NiO NPs possessed greater antimicrobial activity than erythromycin, mainly due to Ni^2+^ positive charge, which is released when NiO interacts with the negative charges present on microbe cell membranes. Ni^2+^ is internalized into the cell membrane and reacts with sulfhydryl groups. Therefore, the activity of synthetase in the microorganism becomes disrupted, thus the cellulose grows through cell division, which in turn leads to microbe death (Rahman et al., 2018).

**Table 10:** Physicochemical characteristics of inorganic NPs.

Silica-based NPs	REF	Functional group	Diameter (nm)	ζ - potential (mV)	Tested uropathogens	Activity
Hydroxyapatite nanoparticles	(Evliyaoǧlu et al., 2011)	–	200	–	*E. coli, Staphylococcus species, P. mirabilis* and *E. cloacae*	Antibiofilm
Sulfur nanoparticles/Catharanthus roseus leaf extract	(Paralikar et al., 2019)	–	53	-9.24	*E. coli, P. mirabilis, P. aeruginosa, K. pneumoniae, E. faecalis* and *S. aureus*	Antibacterial
Tungsten nanoparticles	(Syed et al., 2010)	–	8.1	–	*E. coli*	Antibiofilm
					*E. coli* and *S. aureus*	Antibacterial
Neodymium doped nickel oxide nanoparticles	(Rahman et al., 2018)	Hydroxyl	28	–	*P. mirabilis*	Antibacterial

After the analysis of the reported studies, it becomes evident that the most explored inorganic nanoparticles for UTI treatment are silver-based nanoparticles, alone or in combination with other materials. In this context, the extensive investigation of AgNPs is due to their antimicrobial properties and their ability to combat human pathogen has been showed constantly over the years. The importance of silver in UTI treatment is reflected in its actual use as catheters coating to prevent nosocomial microbial contamination and reduce UTIs as a secondary effect (Politano et al., 2013). Other metals like copper, zinc, gold, and silica share these antimicrobial characteristics with silver, but despite they effectiveness, they have not been approved for human or animal use yet. Other inorganic materials have been also evaluated but with less intensity, which can be related to the difficulty for their approval for human use and thus, their introduction as clinical treatments. Nevertheless, green synthesis is gaining more relevance due to the greater biological and environmental compatibility of these NPs.

Nowadays, inorganic NPs are widely considered as catheters coating, among which AgNPs are already being used to prevent catheter bacterial colonization, while other inorganic materials are currently under investigation. All reported inorganic compounds-based NPs as well as organic NPs, show a tendency to be include in urinary tract devices to prevent biofilm formation and reduce CAUTIs (Meenakumari et al., 2000; Ulett et al., 2007; Syed et al., 2009; Syed et al., 2010; Evliyaoğlu et al., 2011; Agarwala et al., 2014; Prabha et al., 2014; Ahmed et al., 2016; Shafreen et al., 2017; Shalom et al., 2017; Bhargava et al., 2018; Hosseini et al., 2018; Rajivgandhi et al., 2018; Divya et al., 2019; Iribarnegaray et al., 2019; Hosseini et al., 2019; Kanugala et al., 2019). Furthermore, some investigations have exposed that their synthetized NPs could have the potential to be used either as a catheter coating or as a systemic administration drug. However, they need to expand their *in vitro* and *in vivo* evidence for their approval for their clinical use as UTIs treatment (Sivaraj et al., 2014; Li et al., 2014; Qasim et al., 2015; Turcheniuk et al., 2015; Malarkodi and Rajeshkumar, 2017; Al Tameemi et al., 2017; Nsayef Muslim et al., 2017; Lopez-Carrizales et al., 2018; Mishra and Padhy, 2018; Rahman et al., 2018; Tiwari et al., 2018; El-Batal et al., 2019; Rajivgandhi et al., 2019). Other inorganic NPs have also shown a great potential like adjuvants in combination therapy with antibiotics (Bhande et al., 2013; Paralikar et al., 2019). In addition, inorganic NPs are even under the scope as potential systemic therapies for UTI (Huma et al., 2018) (Khoshkbejari et al., 2015).

### Use of Composite Materials

Because of the high resistance that urinary tract pathogens have developed to antibiotics, the development of new effective and potent antimicrobial agents is urgently needed. In this context, composite materials have emerged as novel strategies designed to treat UTIs (**Table 11**). Metal-organic frameworks (MOFs), are organic-inorganic hybrid porous nanostructures, that have been developed to treat infections, since they have the capacity to act as antimicrobial materials, as well as drug carriers (Férey, 2008). The potential of MOFs to combat MDR pathogens is based on the possibility to incorporate different kind of particles into MOFs pores, their metal component, and in their ability to produce physical damage to microbial cells (Wyszogrodzka et al., 2016). In this context, a ZnO-ZIF-8 nanocomposite was evaluated as an antibiofilm agent against four common uropathogens (*E. coli, K. pneumoniae, P. mirabilis*, and *S. aureus*). ZIF-8 is a zeolitic imidazolate framework useful for drug delivery, since its porous features, and for being thermally and chemically stable, and modifiable and pH sensitive (Kaur H et al., 2017). With the MBC assay it was determined that 0.25 µg/mL of ZnO-ZIF-8 compound was able to kill all four microorganisms. The antibiofilm effect was demonstrated by the capability of 2 µg/mL suspensions of ZnO-ZIF-8 to diminish well-established biofilms formed from concentrations of 10^7^-10^9^ CFU/mL to below the limit of detection (BLD). The ZnO-ZIF-8 showed a reduction of 6-8 log, when compared to the control solution, over a 24 h period. Finally, authors of this research prepared ZnO-ZIF-8 embedded silicone elastomers with 2 or 4 w/w % and observed that they effectively eliminated all four microorganisms from the surface after 24 h of exposition, showing their utility to prevent CAUTI (Redfern et al., 2018).

**Table 11:** Physicochemical characteristics of composite materials-based NPs.

Composite materials NPs	REF	Functional group	Diameter (nm)	ζ - potential (mV)	Tested Uropathogens	Activity
ZnO@ZIF8 nanocomposite	(Redfern et al., 2018)	Imidazole	566	–	*E. coli, K. pneumoniae, P. mirabilis* and *S. aureus*	Antibacterial
						Antibiofilm
Silver polytetrafluorethylene nanocomposite	(Zhang et al., 2019)	–	50 - 200 AgNps/150 PTFE Nps	–	*E. coli* and S*. aureus*	Antibacterial
						Antiadhesive
Hydrogel containing copper nanoparticles composite	(Al-Enizi et al., 2018)	Hydroxyl and carboxyl	7	–	*E. coli, K. pneumoniae, P. aeruginosa, P. vulgaris, S. aereus* and *P. mirabilis*	Antibacterial
Tetraether lipids coated silver nanoparticles embedded in a PLGA film loaded with norfloxacin	(Dayyoub et al., 2017)	–	9.2 ± 2.5	–	*S. aereus, S. epidermidis, E. coli, E faecalis* and *P. aeruginosa*	Antibacterial
Silver bearing degradable polymeric nanoparticles of polyphosphoester-block-poly(L-lactide)	(Lim et al., 2015)	–	25 -35	-47	UPEC, *S. aureus*.	Antibacterial
Raman encoded silver coated gold nanorods	(Mahadevegowda et al., 2018)	–	25	-25	UPEC	Antibacterial

In a different research a silver-polytetrafluoroethylene (Ag-PTFE) nanocomposite coating for urinary catheter was developed, with the aim to obtain a synergistic effect between the antibacterial activity of silver and the antiadhesive activity of PTFE. The antibacterial and antiadhesive abilities of these coated catheters were evaluated against two of the most common uropathogens (*E. coli* and *S. aureus).* The anti-adhesion efficacy of Ag-PTFE composite was analyzed by fluorescence microscopy in uncoated, Ag-coated, and Ag-PTFE-coated catheters, determining that Ag-PTFE-coated catheters reduced 55.2-60.3% of *E. coli* adhesion and 49.1-56.5% of *S. aureus* adhesion compared to uncoated and Ag coated catheters, respectively. The antibiofilm ability of the Ag-PTFE-coated catheters was investigated and compared with BARD PTFE-coated Foley catheters and Mediplus all-silicone Foley catheters through a prolonged culturing of 48 hours prior to SEM observation. The SEM results showed that Ag-PTFE-coated catheters diminished the 95.2% biofilm coverage of *E. coli* and the 96.2-97.4% of *S. aureus* biofilm in contrast to the uncoated and PTFE-coated catheters. In addition, with an *in vitro* bladder model authors observed that the Ag-PTFE coated catheters exhibited great anti-infection efficacy against bacteriuria, thus prolonging silicone catheters lifetime from a mean of 6 days to 40 days making these Ag-PTFE coated catheters good candidates to avoid CAUTI (Zhang et al., 2019).

In other investigation the authors synthesized a new nanocomposite formed by hydrogel and copper NPs (HCuNPs). The antibacterial potential of HcuNPs was assessed against *E. coli, K. pneumoniae, P. aeruginosa, P. vulgaris, S. aureus* and *P. mirabilis* strains collected from infected urine samples. To measure HCuNPs and hydrogel antibacterial activity, they applied the agar disc diffusion method. The results indicated that HCuNPs had a superior biocidal activity than parenteral hydrogel in similar conditions giving a ZOIs higher than 16 mm for all the pathogens at a concentration of 5 mg/mL (Al-Enizi et al., 2018).

Dayyoub et al., developed tetraether lipids (TEL)-coated silver nanoparticles distributed in a hydrophobic PLGA film loaded with norfloxacin. TEL are a fundamental part of cell membrane of the archaeon *Thermoplasma acidophilum*; due to the absence of cell wall on the archaeon, TEL are the responsible to provide high thermal and chemical stability (Boyd et al., 2013). These lipids are bound to the glycerol residues by ether bonds, not having double bonds; the latter characteristic provides long-term resistance against biochemical degradation, and oxidative and hydrolytic agents, making these lipids suitable candidates to be used in urinary tract conditions (Dayyoub et al., 2008). In this context, polyurethane (PUR) and silicone sheets have been coated with the polymer films loaded with the antibacterial agents. The antibacterial and anti-encrustation assays were performed using an *in vitro* model of UTI cultures of *S. aureus, S. epidermidis, E. coli, E. faecalis* and *P. aeruginosa*, all of which were cultivated in artificial urine. The results showed that films with TEL-coated Ag NPs effectively inhibited the *in vitro* adhesion of bacteria in about 83%, compared to uncoated sheets (inhibition potential of 48%.) These results were confirmed by scanning fluorescence microscopic images. The results also evidenced that coated films exhibit bactericidal and anti-encrustation effects on their surface (Dayyoub et al., 2017).

In the field of combined materials, silver-bearing degradable polymeric nanoparticles of polyphosphoester-block-poly(L−lactide) were designed for the loading of silver into a hydrophilic shell and/or the hydrophobic core. This “hybrid” delivery system was constructed replacing the polyphosphoester backbone by poly(L-lactide) (PL), a natural lactic acid derived, which is a biocompatible and biodegradable polymer, extensively used for de synthesis of NPs, and possesses high silver loading capacity. Therefore, these NPs were prepared as delivery carriers for Ag-based antimicrobials such as silver acetate (AgOAc) and one of two silver carbene complexes (SCCs). The *in vitro* antimicrobial ability of Ag-NPs and silver compounds was analyzed by MIC determination against 8 UPEC strains. The results indicated the MIC was improved up to 70% with the packaging of the SCCs in the PL-NP-based delivery system, in comparison with the single SCCs, suggesting Ag-PL-NPs would be beneficial in UTIs treatment (Lim et al., 2015).

Other authors explored the use of Raman-encoded silver-coated gold nanorods (GNRs) as scaffolds to attach glycans and engineer multivalent glycan-functionalized GNRs. The objective of these GNRs was to bind UPEC and eliminate them employing a photothermal effect. The antibacterial assay was carried out against an infectious dose of *E. coli* (10^8^ CFU/mL), by employing increasing concentrations of GNRs solutions (from 0 to 0.2 nM). Next, *E. coli*/GNRs dispersions were treated with a NIR laser (808 nm, 1 W/cm^2^) for 15 minutes. The results revealed that in presence of GNRs, the bacteria were eradicated rapidly, due to the efficient photothermal conversion that NIR generated on GNRs (Mahadevegowda et al., 2018).

As summary, composite materials are developed with the objective to have a synergistic effect between inorganic and organic components, making the combination of these kind of materials into nanocomposites a promising alternative for the prevention of CAUTIs, as well as UTIs treatment. Here, we also showed the tendency to develop NPs as catheters coating (Dayyoub et al., 2017; Redfern et al., 2018; Zhang et al., 2019), along with some studies about innovative methods to design and develop therapy for systemic administration (Mahadevegowda et al., 2018). Also, Lim et al., suggested the use of Ag-PL-NPs by direct inoculation intro de bladder, a tendency shown in other investigators with organic and inorganic NPs, due the advantage that local administration represent (Lim et al., 2015). Interestingly, A-Enizi et al., described an innovative application for their HcuNPs, given their great antimicrobial and absorption properties, suggesting their use in diapers, sanitary towels, menstrual pads, and related products (Al-Enizi et al., 2018).

## Future Perspective

Currently, there is a wide range of investigations in relation to new potential candidates to treat and prevent UTIs. It is well known that at Ag-coated catheters can be used to prevent CAUTI with effective clinical response; nevertheless, some studies have shown more efficient particles for the functionalization of catheters and avoid CAUTI by eliminating bacteria and/or inhibiting biofilm formation. Moreover, several of these new nano-compounds have shown no toxicity to human and animal cells. Both, organic and inorganic NPs had exhibited a great potential in the CAUTI prevention, especially used as coating for urinary devices. The benefit for including NPs in these coating is to prevent the infection development, so no biological process will be needed. Thus, the action of these NPs would be local instead of systemic. This represent a huge benefit mainly to avoid pharmacological interactions, considering that hospitalized patients are generally polymedicated. Other technique to achieve the same benefits is intravaginal delivery, which it is limited for women, but it has the advantage that could be used by inpatients and outpatients. However direct bladder delivery should be considered in future investigations, so the same benefits could be achieved for all population. In addition, no cytotoxic effects were revealed with the use of these NPs *in vitro* and *in vivo* (in animal models). In addition, these positive results have led to the idea that NPs could be safe in human clinical trials, and more specific at achieving local drug delivery.

On the other hand, NPs have also been studied as antimicrobial agents themselves, since some of them are able to eradicate bacteriuria caused by both common and MDR uropathogens. In fact, the aim of most of the developed nanocarriers is to enhance antimicrobial properties of molecules that bacteria have already developed a mechanism of resistance. In this context, metal NPs are very useful because, since they do not target metabolic pathways, they do not trigger bacterial resistance mechanisms. Nevertheless, the synthesis of metal NPs includes the use of toxic elements that makes their safety questionable and represent potential environmental problems. In this context, green synthesis of inorganic NPs appears to be the solution; using this strategy, inorganic nanoparticles would have the same opportunities as organic nanoparticles to be considered as powerful candidates for bio and nano medicine to help in the finding of new potent antimicrobial agents. Furthermore, the use of plants as reductor agents will be beneficial in the synthesis of NPs because the medicinal compounds of the plant could be included into the NPs enhancing their properties. It is also important to consider that the investigation with biocompatible materials is an issue that requires faster progression in the application of nanotechnology to treat this pathology. Unfortunately, very few studies included the analysis of pharmacokinetic parameters being this the first step towards clinical studies. It is therefore important to start including pharmacokinetic and pharmacodynamic studies in future investigations on the inclusion of nanosystems as new treatment strategies for UTIs and CAUTIs. Finally, we have to consider that the use of new medicines in humans need a large validation process, thus the use of already approved materials might be the solution to accelerate the possibility to use of nano-compounds in the treatment and prevention of UTIs, and cover the actual demands.

## Author Contributions

SS summarized the literature and drafted the manuscript. JM, JC-F, and NN revised and edited the manuscript. SS and JM designed the content and structured the manuscript. All authors contributed to the article and approved the submitted version.

## Funding

This work was funded by Regular FONDECYT Project 1181689, ANID/PIA/ACT192144, ANID/PCI/REDI170653, FONDAP Project 15130011, National Doctoral Scholarship 21201831, all granted by Chilean National Agency for Research and Development (ANID).

## Conflict of Interest:

The authors declare that the research was conducted in the absence of any commercial or financial relationships that could be construed as a potential conflict of interest.
